# Thermodynamics 2.0: Bridging the natural and social sciences

**DOI:** 10.1098/rsta.2022.0275

**Published:** 2023-08-07

**Authors:** Ram Poudel, Jon McGowan, Georgi Y. Georgiev, Emmanuel Haven, Umit Gunes, Hongkun Zhang

**Affiliations:** ^1^ International Association for the Integration of Science and Engineering (IAISAE); ^2^ Appalachian State University, Boone, NC, USA; ^3^ University of Massachusetts, Amherst, MA, USA; ^4^ Assumption University, Worcester, MA, USA; ^5^ Memorial University of Newfoundland, St John's, Canada; ^6^ Yildiz Technical University, Istanbul, Turkey

**Keywords:** energy, power, evolution, economics, human society, unity of science

## Abstract

Thermodynamics is a universal science. The language of thermodynamics is energy and its derivatives such as entropy and power. The physical theory of thermodynamics reigns across a full spectrum of non-living objects as well as living beings. In the traditions of the past, the dichotomy between matter and life resulted in the natural sciences studying matter while the social sciences focused on living beings. As the state of human knowledge continues to evolve, anticipating the sciences of matter (natural science) and of life (social science) becoming unified under a single overarching theory is not unnatural.

This article is part of the theme issue ‘Thermodynamics 2.0: Bridging the natural and social sciences (Part 1)’.

## 1. Introduction

Thermodynamics is a universal science. The language of thermodynamics is energy and its derivatives such as entropy and power. The physical theory of thermodynamics reigns across a full spectrum of non-living objects as well as living beings. In the traditions of the past, the dichotomy between matter and life resulted in the natural sciences studying matter while the social sciences focused on living beings. As the state of human knowledge continues to evolve, anticipating the sciences of matter (natural science) and of life^1^ (social science) becoming unified under a single overarching theory is not unnatural.

This theme issue is an effort in that direction, one where we consider the unity of science within the language of thermodynamics. We have included 11 articles in this theme issue dealing with theoretical and applied advances for bridging the natural and social sciences with emphasis on methods sourced from thermodynamics and quantum theory.

A universal science would recognize no boundary between non-living and living. One open question is the nature of relationship between natural and social scientific studies. Our understanding of science can have many limitations that follow our culture and understanding of nature. Just as our brain is divided in two with the right and left hemispheres, so are our sciences divided in two with the natural and social sciences: one facing the West and the other facing the East, to paraphrase Iain McGilchrist [1]. The difference between the two sciences is profound—two whole, coherent, but incompatible ways of understanding the nature of which human beings are a part. To claim that we have reached a higher level of comprehension and consciousness for enacting the foundation of integrated science would be quite supreme. Nevertheless, we believe that thermodynamics may be able to provide such an ontological foundation and help bridge the natural and social sciences toward their unity. Clearly, more work remains to be done.

If you want to find the secrets of the universe, think in terms of energy, frequency, and vibration, Nikola Tesla said some time ago. *Thermodynamics 2.0* is an attempt along this line of thought to discover the secrets of living beings and their connection with that which is ‘non-living’ in the universe. By design, *Thermodynamics 2.0* bridges the natural and social sciences by using constructs and ideas, such as temperature, energy, power and more. These thermodynamics-based constructs are independent of the materials under study, while at the same time know none of the academic boundaries we created about three centuries ago. Attempts to divide science into two ought to be regarded with much suspicion, subtilizing any more would bring more disadvantages than it is worth [2].

We can differentiate systems into closed and open systems. In an open system, both energy and matter cross system boundaries. A science of open systems aims to explain the increasing order of complexity and organization we have observed in nature since the Big Bang. This science awaits new improvements and discoveries. Open systems can structure themselves, evolve and live. Human beings are an open system and also a pinnacle of evolution. Carl Sagan and Eric Chaisson (Harvard University) coined the term cosmic evolution to explain all of the self-organization processes in open thermodynamic systems from the Big Bang until today, leading from pure energy to our complex society [3]. The cosmic evolution hypothesis strives to integrate the large with the small, the near with the far, and the past with the present, all into a unified whole. This theme issue contributes to a unified science that encompasses both open and closed systems. Researchers apply the principles of thermodynamics to help better understand open and evolving systems and to contribute to developing the basic principles.

Is thermodynamics a science on its own? Many physicists like to boast thermodynamics as an exclusive branch of physics. However, numerous engineers and other researchers disagree with such statements [4]. In addition, J. H. Lienhard (University of Houston) has wondered if thermodynamics really is a science [4], Lienhard probably meaning physics by saying a science. Some sporadic efforts have occurred to expand and enlarge the scope of thermodynamics. Libb Thims at the Encyclopedia of Human Thermodynamics has summarized about 12 founding schools of thermodynamics. In recent times, mechanical engineers such as Joseph Keenan (MIT) and Adrian Bejan (Duke University) have also pushed the boundaries of thermodynamics on various fronts.

Thermodynamics preceded quantum mechanics. If we look at the structure of the time-dependent Schrödinger equation, it is difficult to judge whether quantum mechanics is based on engineering thermodynamics or the other way around. Thermodynamics is capable of linking various branches of physics [5,6], and thus its consistency with thermodynamics unsurprisingly led to Planck's Law and the dawn of quantum theory. One author claims that quantum mechanics inserts dynamics into thermodynamics [7]. This evolving field of study is known as quantum thermodynamics. However, one does not have to follow quantum mechanics to insert greater dynamics into thermodynamics the definition of power as the rate of change of energy is enough to bring temporal and dynamic aspects into a thermodynamic analysis. Building on circular reasoning like this, scholars often try to establish the emergence of thermodynamic laws based on quantum mechanics [7], but the laws of thermodynamics are fundamental and much broader in scope. Einstein opined the following about thermodynamics: ‘It is the only physical theory of universal content, which I am convinced, that within the framework of applicability of its basic concepts will never be overthrown’.

When we talk about thermodynamics, we need to be mindful of the ensemble and level of description we are pursuing. Thermodynamics is a science of the forest and not just the trees [4]. In science, we normally infer these levels by the three prefixes of micro-, meso- and macro-. Sociologists also study society on three distinct levels [8]. Macro-level sociological analysis is an examination of society as a whole that looks at the broad systems, institutions, hierarchies, and patterns that shape a society. Meso-level sociological analyses study certain parts of society, such as specific groups, communities, or organizations. One-to-one interactions between individuals are studied under micro-level analyses and include studying people's behaviour during negotiations, confrontations, and everyday conversations. Thermodynamics is an odd science at the crossroads of life and non-life. The emergence and evolution of life are indeed based on physics but remain beyond any physics we thus far come to know [9]. This gap and inconsistency in the human knowledge of science has been a motivation for the contributions of many authors in this volume.

Thermodynamics is a science of macro-level description at best and connects various levels of description through bridges such as the Sackur–Tetrode equation [10], which presents an expression for the absolute entropy that helps relate the classical definition to that from quantum mechanics.

In general, a set of differential equations model a dynamic system. The widespread misconception also exists that thermodynamics can tell us nothing about mechanisms, dynamics, and evolution. Scholars are found who still think of thermodynamics as an energy book-keeping system, including some who have spent decades in science [11]. This mindset has hindered physics from realizing its full potential in the domain of life. How did cooperative behaviour evolve [12]? Unfortunately, this question is still at large in science. Nonetheless, we have recently started thinking about the Physics of Life and concerting efforts toward understanding the rules of life. The National Science Foundation has started supporting its larger ideas under the theme of Understanding the Rules of Life [13].

## 2. The papers: *Thermodynamics 2.0* | Part I

The theme of *Thermodynamics 2.0*: *Bridging the Natural and Social Sciences* includes articles that use the three languages of energy, entropy, and information, as well as their proxies (e.g. power, networks) in the social sciences, with the aim of erecting a more unified system of human knowledge. One goal of this topical issue is to illuminate the scope and reach of thermodynamics. We are attempting to encompass a set of diverse viewpoints and alternative theories with this special issue. You may be amazed to see elaborate questions that can be formulated and answered within the framework of thermodynamics. We leave it to future generations to decide what makes better sense to them.

We start this theme issue with the article by Arto Annila [14] (University of Helsinki), who in it illuminates the universal nature of thermodynamics. Arto conjectures on quanta of light as the basic building blocks of everything in order to come up with philosophical arguments as to why thermodynamics makes sense across the disciplines of the natural and social sciences alike. This article is followed by two articles that attempt to illuminate the unity of science. A unified theory by Ram Poudel [15] (Appalachian State University) generalizes Newton's Third Law of Motion to connect life with non-life. The unified framework encompasses social field theory as it pertains to the laws and rules of life. This theory and the underlying equations of motion and change have direct correspondence to the science of matter. Rod Swenson [16] (University of Connecticut) addresses the incommensurability of physics, life and mind with the law of maximum entropy production as a basis for his grand unified theory. This theory attempts to unite the river of physics that ‘flows down’ to disorder with the river of life and mind that ‘flows up’ to higher states of order.

The thermodynamics of organisms and life is examined by Dilip Kondepudi and colleagues [17] (Wake Forest University) and Terrence Deacon *et al*. [18] (University of California Berkeley). Both Dilip and Terry build their argument on dissipative structures introduced first by Ilya Prigogine. Organisms are dissipative systems but machines are not; organisms exhibit intentionality or goal-directed behaviour—known also as teleology. The physical basis of exploration of this important aspect of organism is thermodynamics. Terry explains teleological causality in organisms in terms of a codependent structure. Two or multiple self-organizing processes linked by a shared substrate can develop toward a self-sustaining targeted state. This natural model of teleological causation, however, is applicable to the far-from-equilibrium dissipative dynamics of self-organized processes.

Peter Van [19] (Wigner Research Centre for Physics) argues thermodynamics as a stability theory with the viewpoint that thermodynamics is a part and outcome of a general dynamical theory. This is one reason why the physical concepts of thermodynamics are universal and therefore help formulate dynamical theories of any systems in the social and natural sciences.

We then move to the realm of quantum physics in order to bridge the natural and social sciences. Vikram Athalye (Cummins College) and Emmanuel Haven (Memorial University) [20] use the notion of causality and the idea of an ensemble to assert quantum physics-analogous models of social reality. An argument in favour of this assertion is supplied for two social situations modelled by Markov processes. Cal R. Abel [21] (StatisticalEconomics.org; Signal Power and Light, Inc.) provides new perspectives on the utility and value in using quantum formalism. Von Neumann entropy and von Neumann–Morgenstern utility are established as being equivalent, with the Hamiltonian operator representing value. Andrei Khrennikov [22] (Linnaeus University) expands the social laser theory with the notion of an *infon*—a social energy quantum carrying coarse-grained information content. This theory perceives humans as atoms' analogues, namely as social atoms that absorb and emit *infon*s. The theory is also linked to decision-making models.

Umit Gunes [23] (Yildiz Technical University) examines the interactions between social sciences and energy research using publications in the Scopus database. Publications on sustainability, climate change, innovation, urban development and alternative energy were found to be more prominent for sustainability than other more technical subjects.

Adrian Bejan [24] (Duke University) makes an unsolicited attempt to address the open questions [25] of *Thermodynamics 2.0* using his pet theory known as Constructal Law. Adrian's article underpins Constructal Law as the universal principle of all biological, geophysical, social, and technological evolution.

A relationship exists between physics and thermodynamics, as exemplified by statistical approaches [6,26]. The relationship is not reciprocal, as the practitioners of science value this relationship asymmetrically. Engineers value thermodynamics more favourably than physicists might, and this value judgment from engineers has a legacy and logic. Thermodynamics came into being after we had discovered steam engines because people felt the need to explain just how their new engines worked.

We hope this two-part theme issue of *Thermodynamics 2.0* will be able to contribute toward the unity of science, energy, economics and evolution. The fact that engineers in the twenty-first century study above and beyond engines has become increasingly evident. We should also spend more time understanding and explaining how humans and human society work. *Thermodynamics 2.0* strives toward a better understanding of life, humans and human society, as well as their interconnection to the material world. Energy makes a human society, and power relationships make or break a society.

## Data Availability

This article has no additional data.

## Footnotes

1 We use the general term of social science instead of the more specific term of life science.
